# Sleep improvement after hip arthroplasty: a study on short-stem prosthesis

**DOI:** 10.1007/s00264-019-04375-1

**Published:** 2019-07-27

**Authors:** Josef Hochreiter, Harald Kindermann, Mattiassich Georg, Reinhold Ortmaier, Marian Mitterer

**Affiliations:** 1 Department of Orthopedic Surgery, Ordensklinikum Barmherzige Schwestern Linz, Vinzenzgruppe Center of Orthopedic Excellence, Teaching Hospital of the Paracelsus Medical University, Salzburg, Austria; 2 Department of Marketing and Electronic Business, University of Applied Sciences Upper Austria, Campus Steyr, Wels, Austria; 3 Trauma Center Graz, Graz, Austria; 4 Institute for Sports Medicine, Alpine Medicine and Health Tourism (ISAG), Tirol Kliniken GmbH, Innsbruck and UMIT, Hall in Tirol, Austria; 5 Department of Orthopaedics and Traumatology, Paracelsus Medical University Salzburg, Müllner Hauptstrasse 48, 5020 Salzburg, Austria

**Keywords:** Total hip arthroplasty, Short-stem hip arthroplasty, Sleep, Pain, Hip osteoarthritis

## Abstract

**Purpose:**

The purpose of this study was to evaluate sleep disturbance prospectively before and after short-stem hip arthroplasty.

**Methods:**

A prospective study on 25 patients undergoing a primary unilateral total short-stem hip replacement was conducted. Patients were observed for six months. To evaluate the sleep quality and daytime sleepiness, the Pittsburgh Sleep Quality Index and Epworth Sleepiness Scale were used. To assess the general physical health status, we used the Short Form 36 Health Survey (SF-36). Pain was recorded on a visual analog scale.

**Results:**

The physical health status of the patients improved significantly (*p* < 0.05) during the six month follow-up period in seven out of nine categories. During the first post-operative week, the sleep quality stayed on an equal level to the pre-operative state, following a steady improvement over the next months (6 months *p* = 0.00). The daytime sleepiness showed a significant improvement during all the follow-ups (6 months *p* = 0.00). Pain decreased significantly from baseline to six months post-operatively (*p* = 0.00). There was no correlation between pain and sleep quality or pain and daytime sleepiness.

**Conclusion:**

According to our results, patients undergoing short-stem total hip arthroplasty can expect a 50% improvement of sleep quality and physical function six months after surgery.

## Introduction

Hip osteoarthrosis is a common condition, and approximately one-third of adults in the USA and up to 23% of the adults in Europe are affected [1, 2]. Women have a higher incidence of hip osteoarthrosis than men, at a ratio of 2:1 [3].

According to the World Health Organization, 80% of adults with osteoarthrosis have limitations in movement, and 25% cannot perform their major daily activities of life [3]. Patients with hip osteoarthrosis often describe a reduced social function as well as worse sleep quality and an increase in sleep medication use [4–6].

A reduced sleep quality correlates with increased night pain and severity of osteoarthrosis [7].

The current gold standard for hip osteoarthrosis is a total hip replacement [8]. For younger patients with hip osteoarthrosis, the short-stem hip prosthesis is a new method that promises reduced surgical trauma as well as faster post-operative recovery compared with standard-stem prosthesis [9, 10].

After total hip replacement, studies have shown a distinct increase in quality of life and energy/vitality (SF-36) as well as reduced daily and night pain [11–13].

Previous studies observed sleep quality, pain, and physical function in patients undergoing hip and knee arthroplasty up to 24 months after surgery [11, 12, 14]. Their results found improved sleep quality and decreased pain after arthroplasty but with an initial worsening and weeks of delay [15].

Prospective studies are lacking, and no information exists about sleep disturbance after short-stem hip arthroplasty.

This study intended to determine the sleep quality and daytime sleepiness in a prospective evaluation during several defined follow-up time points (up to 6 months post-operative) to identify correlations and to improve the preoperative information giving to patients.

## Materials and methods

A prospective cohort study was conducted on patients with primary unilateral hip arthroplasty from January 2016 to August 2016.

We included patients undergoing primary unilateral short-stem hip arthroplasty. The mean age of the study collective was 68.0 years. The indication for surgery was primary osteoarthritis on the affected hip. The inclusion criteria were patients older than 18 years who agreed to and were able to complete the pre- and post-operative surveys.

The exclusion criteria were patients who presented any other indication than primary osteoarthritis such as revision surgery, patients with infectious complications after surgery, history of pre-existing sleep disorder, history of supplemental sleeping aids and patients with dementia or mild cognitive impairment as well as patients with sleep apnea syndrome.

Overall, 25 patients were included. The details of the patient demographics are shown in Table 1. In all the patients, the PSQI, Epworth Sleepiness Scale, SF-36, and VAS were obtained pre-operatively, one week, three weeks, six weeks, three months, and six months post-operatively.

**Table 1:** Demographics (age, sex, BMI) of the study population

	Patients (*n* = 25)
Age (year)	68.0 ± 9.7
Body mass index (kg/m^2^)	26.5 ± 5.0
Sex female	19 (76.0%)

Ethical approval was obtained, and all the patients signed an informed consent before participating in this study.

Patients received no supplemental sleeping aids during the study period and no prescription afterward. The average hospital stay was six ± two (range, 3–8) days.

The current health status was evaluated using the Short Form 36 Health Survey (SF-36) [16]. This questionnaire measured eight multi-item variables using 36 self-rating questions. For each variable, the item scores were coded, summed, and transformed onto a scale from 0 (worst possible health status) to 100 (best possible health status) [16].

All the 25 patients had to fill out the SF-36 pre-operatively and six months after the surgery.

For assessing the sleep disturbance and quality, we used both the Pittsburgh Sleep Quality Index (PSQI) and the Epworth Sleepiness Scale (ESS).

The PSQI is a self-rated questionnaire that collects nineteen individual items to measure sleep quality and disturbances. The seven  categories were subjective sleep quality, sleep latency, sleep duration, habitual sleep efficiency, sleep disturbances, use of sleeping medication, and daytime dysfunction. These categories were summed up to create a total score with a maximum of 21. A higher score indicates worse sleep dysfunction, and a score greater than five indicates poor sleep quality. All the study patients were asked to fill out the PSQI pre-operatively and during the post-operative checkups in the following intervals: one week, three weeks, six weeks, three months, and six months. Patients who could not attend every checkup were called and completed the survey over the telephone.

To assess the daytime sleepiness, we used the ESS. The ESS is a validated questionnaire that quantifies the severity of daytime sleepiness [17]. An ESS score higher than ten indicates significant sleepiness. The ESS questionnaire was conducted preoperatively as well as postoperatively after one week, three weeks, six weeks, three months, and six months.

To assess the pain pre-operatively and after the surgery, we used a visual analog scale (VAS) from 0 to 10.

Furthermore, demographic data such as sex, age, and body mass index were collected to analyze subsidiary factors to sleep disturbance.

### Statistical analysis

Statistical analysis was performed using SPSS for Windows, version 25. The categorical variables are expressed as frequency and percentage, whereas ordinal variables are represented as the median and interquartile range (25th percentile; 75th percentile). The Wilcoxon signed-rank test was used to compare the nonparametric time-dependent variables. Differences were considered statistically significant if *p* < 0.05.

### Surgical technique

For all the patients, an anterolateral, modified Watson-Jones approach was used. A cement-less, monobloc short-stem (Optimys; Mathys, Bettlach, Switzerland) and a cement-less press-fit, monobloc vitamin E–enriched HXLPE cup coated with titanium particles (RM Pressfit vitamys; Mathys, Bettlach, Switzerland) were used. All the patients received full weight-bearing ambulation under surveillance of physiotherapy and started using crutches immediately on post-operative day 1 with an initial restriction of flexion at 90° for one week.

### Rehabilitation

Full weight-bearing using crutches under surveillance of a physiotherapist was allowed immediately on post-operative day 1. Functional active and passive motion, with initial restriction of 90° of flexion for one week was allowed. After four to six weeks, all patients underwent inpatient or outpatient rehabilitation program five days a week for two to three weeks.

## Results

A significant improvement could be detected in seven out of the nine subcategories of the SF-36. Only the subcategories Social Function and Health Perceptions showed nonsignificant results due to missing figures.

The pre-operative mean physical function was 38.3% ± 24.1%, and the energy/vitality was 47.4% ± 18.7% in adults with unilateral hip osteoarthrosis. During the following six months, the post-operative physical function improved to 61.8% ± 1.5% (*p* = 0.00), and the energy/vitality increased to 60.4% ± 15.6%. A Wilcoxon signed-rank test revealed that this improvement was significant, *z* = − 4.29, *p* = 0.00, with a large effect size (*r* = 0.88) (Table 2).

**Table 2:** Change in SF 36, PSQI, ESS, and VAS after short-stem hip arthroplasty during the study at the 6 checkups

	Pre-operative	1 week	3 weeks	6 weeks	3 months	6 months
SF-36						
Physical functioning	38.3%	–	–	–	–	61.8%
Role physical	25.0%	–	–	–	–	60.0%
Pain	29.0%	–	–	–	–	84.5%
Change in health	31.0%	–	–	–	–	72.0%
Energy/vitality	47.4%	–	–	–	–	60.4%
Social functioning	72.5%	–	–	–	–	80.5%
Role emotional	65.2%	–	–	–	–	89.4%
Mental health	73.9%	–	–	–	–	77.9%
Health perceptions	65.0%	–	–	–	–	63.0%
PSQI	9.6 ± 3.9	9.5 ± 4.4	6.3 ± 2.9	5.4 ± 2.8	5.8 ± 2.8	4.3 ± 2.0
Duration of sleep	1.0 ± 1.1	1.0 ± 1.2	0.6 ± 1.0	0.4 ± 0.7	0.7 ± 0.9	0.0 ± 0.0
Sleep disturbance	1.7 ± 0.5	1.6 ± 0.5	1.3 ± 0.5	1.2 ± 0.4	1.2 ± 0.4	1.2 ± 0.4
Sleep latency	1.8 ± 0.8	1.8 ± 0.8	1.3 ± 1.0	1.3 ± 1.1	1.3 ± 0.9	1.1 ± 1.0
Day dysfunction	1.1 ± 0.6	1.0 ± 0.9	0.7 ± 0.7	0.4 ± 0.6	0.6 ± 0.7	0.5 ± 0.7
Sleep efficiency	1.5 ± 1.2	1.5 ± 1.2	0.8 ± 0.8	0.7 ± 0.9	0.8 ± 1.1	0.3 ± 0.6
Sleep quality	1.4 ± 0.7	1.4 ± 0.8	1.1 ± 0.5	0.9 ± 0.5	0.7 ± 0.6	0.8 ± 0.6
Medication	0.7 ± 1.2	1.0 ± 1.4	0.7 ± 1.2	0.8 ± 1.3	0.7 ± 1.2	0.7 ± 1.2
ESS	7.4 ± 5.6	5.8 ± 4.8	4.3 ± 2.6	4.5 ± 2.8	3.9 ± 2.8	4.2 ± 3.6
VAS pain	5.5 ± 1.5	3.6 ± 1.7	2.1 ± 1.8	0.8 ± 0.9	0.2 ± 0.5	0.1 ± 0.3

**Fig. 1:**
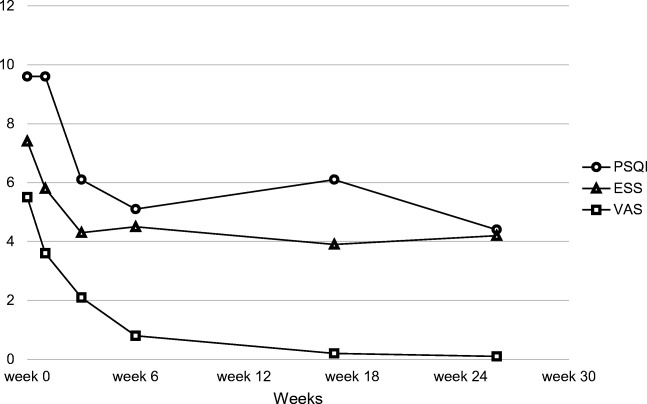
Pre-operative and post-operative PSQI, ESS, and VAS after short-stem hip arthroplasty. Pre-operative and post-operative PSQI, ESS, and VAS during the period of record. The diagram shows a clear trend of increase in sleep quality (PSQI), as well as a decrease in daytime sleepiness (ESS) and pain (VAS) compared with baseline

The average pre-operative PSQI score was 9.1 ± 3.9 in the 25 participating patients. The PSQI score increased slightly over the first post-operative week following a nearly uninterrupted decrease over the next weeks and months. Six months after surgery, the mean PSQI was 4.3 ± 2.0 (*z* = − 3.22, *p* = 0.00, *r* = 0.80), which indicates a normal sleep (Fig. 1).

The greatest improvements from the pre-operative period until six months after surgery were made in the categories of subjective sleep quality, sleep latency, sleep duration, habitual sleep efficiency, and daytime dysfunction.

The ESS dropped significantly from a pre-operative value of 7.4 ± 5.6 to 4.2 ± 3.6 (*z* = − 3.61, *p* = 0.00, *r* = 0.72) during the following weeks. The improvement of daytime sleepiness was comparable with the PSQI results.

VAS score decreased continuously from baseline 5.5 ± 1.5 over all the points and checkups during the six months post-operative follow-up until 0.1 ± 0.3 (*z* = − 4.43, *p* = 0.00, *r* = 0.89).

There was no significant correlation between the VAS and PSQI and between the VAS and Epworth sleepiness scale.

## Discussion

Sleep disorder in patients with osteoarthrosis in the hip or other joints is a common condition [18]. Pain is an essential parameter in patients with sleep disturbance and osteoarthrosis [8, 18, 19]. Multiple studies have demonstrated that primary hip arthroplasty improves patients’ quality of life as well as sleep disturbance after surgery. Most of the previous studies focused on the hospitalization period and the first weeks after surgery (up to 3 months) [8, 18, 20].

This study was conducted to observe changes in sleep disorder in patients with unilateral hip osteoarthrosis undergoing a primary short-stem hip replacement. The goal was to collect patient data from a pre-operative baseline period until reintegration to normal daily activities six months after surgery.

The patients reported poor sleep quality at the beginning of the study, which is comparable with other studies that showed increased pain and lack of sleep in patients with hip osteoarthrosis [7, 11].

As shown in other studies, sleep quality decreases in the first week after surgery to increase again in the following weeks to months [15]. Other studies have already established poor sleep quality at baseline levels up to one month after surgery in patients with total hip replacement [21]. In our study, sleep quality according to PSQI stayed the same level at the first week after surgery following a substantial improvement in all categories, except sleep medication abuse, over the following weeks. Even three months after surgery, there was still improvement relating to PSQI. The Epworth sleepiness scale, which investigated the daytime sleepiness as well as the VAS pain scores, improved substantially after the first post-operative week. The Epworth sleepiness scale did not change significantly after the first post-operative week, while the VAS showed a continuous drop after surgery and was never above the baseline level during the study [22].

Regarding the quality of life, our study suggests a significant improvement after hip arthroplasty in patients with osteoarthrosis. Pre-operatively, the participants had poor SF-36 scores in physical functioning, general health, and body pain. This correlates to pre-existing studies suggesting reduced health and quality of life in patients with chronic osteoarthrosis [12]. After surgery, the greatest improvements were in the scores for physical functioning, role physical, general health, and body pain [11, 14]. Moreover, improvements in social function, vitality, and mental health were observed. These findings suggest that improved physical function and general health after surgery in patients with chronic osteoarthrosis have a mild impact on nonphysical related limitations [12, 23].

The limitations of this study include a small cohort. A relatively unequal spreading in gender, the female to male ratio was 76%:24%, the preliminary exclusion criteria of patients with revision hip arthroplasty and patients using sleeping medication. Another limitation of this study is the absence of a control group, especially of a standard-stem group, which permits to draw definitive conclusions about differences in sleep recovery between standard and short stems.

However, the difference in the quality of life after short-stem and standard-stem hip arthroplasty has already been reviewed in several studies that suggest similar functional outcome with slight benefit for short stem prosthesis in younger patients due to faster post-operative recovery as well as less stress shielding [9, 10, 24, 25]. A supplement reduction of thigh pain using short-stem hip arthroplasty might has an effect on sleep improvement, but existing studies show no significant difference compared with conventional standard-stem hip arthroplasty [26].

Nevertheless, a prospective, randomized control trial including two groups of short stems and standard stems would help to show if there are real differences of sleep recovery between standard- and short-stem hip arthroplasty.

This study demonstrates a post-operative decrease or steady state of sleep quality in patients with unilateral short-stem hip arthroplasty. However, in the following weeks and months, patients can expect substantial improvement regarding sleep quality, daytime sleepiness, quality of life, and pain. This information can help to shape patients’ expectations pre-operatively and reducing direct post-operative frustration due to potential sleep improvement even three months after surgery.
